# Timing of Tocilizumab Administration Under the Guidance of IL-6 in CAR-T Therapy for R/R Acute Lymphoblastic Leukemia

**DOI:** 10.3389/fimmu.2022.914959

**Published:** 2022-06-21

**Authors:** Yinqiang Zhang, Fen Zhou, Zhuolin Wu, Yingnan Li, Chenggong Li, Mengyi Du, Wenjing Luo, Haiming Kou, Cong Lu, Heng Mei

**Affiliations:** ^1^ Institute of Hematology, Union Hospital, Tongji Medical College, Huazhong University of Science and Technology, Wuhan, China; ^2^ Hubei Clinical Medical Center of Cell Therapy for Neoplastic Disease, Wuhan, China; ^3^ Department of Pediatrics, Union Hospital, Tongji Medical College, Huazhong University of Science and Technology, Wuhan, China

**Keywords:** chimeric antigen receptor T cell, cytokine release syndrome, acute lymphoblastic leukemia, tocilizumab, interleukin-6

## Abstract

Chimeric antigen receptor T (CAR-T) cells targeting CD19 have achieved great clinical responses in patients with relapsed or refractory (R/R) acute B lymphoblastic leukemia. However, severe adverse events such as cytokine release syndrome (CRS) and immune effector cell-associated neurotoxicity syndrome restrict it to further application. Tocilizumab is the corner stone for the treatment of severe CRS. It has been used to treat mild CRS in recent years, whereas some statistical supports clarifying the suitable timing of its administration are lacking. Sixty-seven patients with B-cell acute lymphoblastic leukemia (B-ALL) were treated with CD19-CART and enrolled in the study, of which 33 patients received Tocilizumab. Application of Tocilizumab in patients with grade 2 CRS in American Society for Transplantation and Cellular Therapy (ASTCT) criteria can significantly shorten the duration of CRS without affecting side effects and long-term efficacy. However, a number of patients still developed severe CRS with early use of Tocilizumab, indicating the significance of the introduction of clinical laboratories to assist medications. Statistically, patients with less than fourfold increase in IL-6 levels had a higher incidence of severe CRS after receiving Tocilizumab (37.5% versus. 0%, p=0.0125), which provided a basis for refining CRS intervention strategies under the guidance of IL-6.

**Clinical Trial Registration:**

www.clinicaltrials.gov, NCT02965092 and NCT04008251

## Introduction

Chimeric antigen receptor T cell (CAR-T cell) is an emerging and promising therapy for hematologic malignancies. Up to now, five commercialized CAR-T products have been approved by the Food and Drug Administration. CAR-T cells targeting CD19, which is broadly expressed by B-cell malignancies, had shown amazing results in several clinical trials (1–4) with a complete remission rate up to 90% (3). Although excellent efficacy of CAR-T cell has been presented, lethal side effects represented by cytokine release syndrome (CRS) and immune effector cell-associated neurotoxicity syndrome (ICANS) could not be overlooked.

CRS is the most common toxicity of CAR-T therapy with classic symptoms of pyrexia, fatigue, hypotension, and hypoxicemia. There are several grading systems (5–7) for the classification of CRS associated with CAR-T therapy, among which the American Society for Transplantation and Cellular Therapy (ASTCT) grading system is now adopted in most research and clinical management due to its simplicity and ease of implementation. The mechanism of CRS is still not fully understood, but what is certain is that in the complex inflammatory cascade, immune cells such as macrophages and the various inflammatory factors they release play a major role. The guideline (8) recommends the use of Tocilizumab, corticosteroids, and life-support treatments for CRS, but the ideal timing for the launch of these treatments is still under debate. In recent years, there have been numerous studies exploring the effects of corticosteroids on CAR-T therapy. Several studies (9–11) have demonstrated that the early use of corticosteroids did not affect the amplification of CAR-T, and the long-term efficacy of immunotherapy in lymphoma and leukemia. However, Strati et al. (12) found that higher a cumulative dose and prolonged and early use of corticosteroids were all associated with significantly shorter overall survival.

Tocilizumab is an IL-6 antagonist that can precisely block interleukin-6 (IL-6), which has been shown to be associated with severe CRS (1, 13). It is used in rheumatologic diseases and was approved by FDA to treat CAR-T-associated CRS in patients older than 2 years old (14). Tocilizumab did not influence the proliferation, persistence, and efficacy of CAR-T and did not present severe side effects (2), so there are few studies on the safety and efficacy of Tocilizumab in early administration. Caimi et al. and Banerjee et al. reported that prophylactic Tocilizumab administered 1 h before CAR-T cell infusion or early Tocilizumab given ≤12 h after CRS onset was beneficial for the domination of CRS in non-Hodgkin lymphoma and multiple myeloma patients, respectively (15, 16). However, one preliminary result suggested that the prophylactic use of Tocilizumab did not inhibit or even increase the incidence of ICANS in NHL (17). Studies aiming for the early treatment of Tocilizumab are lacking in patients with leukemia. In the published papers, the onset of Tocilizumab mainly depends on the grading systems. Whether to introduce the quantitative biomarkers into management strategy and optimize the window period of Tocilizumab have certain significance for further improving the safety of CAR-T therapy.

For the purposes described above, we conducted a retrospective analysis of 67 patients with B-ALL to determine the timing for treatment according to patients’ symptoms and serum biomarkers and established a flow chart of early intervention strategies for patients with CRS.

## Methods

### Patients and Infusion of CAR-T Cells

Complying with the inclusion and exclusion criteria in two clinical trials (NCT02965092, NCT04008251), 75 patients with R/R B-ALL were enrolled in this study. Patients received lymphodepleting chemotherapies following the infusion of murine or humanized CD19 CAR-T cells. The manufacturing and infusion of CAR-T cells were in accordance with a previous article (18). Informed consent in accordance with the Declaration of Helsinki were provided by every patient before participation. Apart from the eight patients not meeting the inclusion criteria, 67 patients were included in this study.

### Definitions and Management of CRS

CRS was graded according to the ASTCT consensus, which has a concise interpretation with the following symptoms: grade 1, temperature ≥38°C; grade 2, fever with hypotension or/and hypoxia, not requiring vasopressors or high-flow nasal cannula; grade 3, administration of one vasopressors or/and supplemental oxygen like high-flow nasal cannula and facemask with fever; and grade 4, administration of more than one vasopressor or/and positive pressure respiratory support system.

We further defined grade 1–2 as mild CRS and grade 3–4 as severe CRS. Fever unattributable to any other cause was the onset of CRS. Since temperature can be influenced by administration of corticosteroids, the end of CRS was associated with disappearance of fever and declined cytokines.

Apart from symptomatic treatments such as antipyretic actions, oxygen therapy, and vasopressors, Tocilizumab and corticosteroids are common drugs in the treatment of CRS. Patients who developed persistent fever unrelated to infection and did not respond to antipyretic drugs received Tocilizumab. If symptoms persisted or progressed after two doses of Tocilizumab, corticosteroids such as dexamethasone and methylprednisolone were considered.

### Assessment of ICANS and Infections

Neurological symptoms were evaluated and graded according to ASTCT consensus. Headache as a separate symptom was not considered neurotoxic. Corticosteroids were used as first-line agents for neurotoxicity. The diagnosis of infection was based on clinical symptoms and etiological examinations. The classification criteria were in accordance with Common Terminology Criteria for Adverse Events v4.0.3 (CTCAEv4). Severe infection was defined as grade 3–4 infection in which intravenous fluids were required.

### Assessment of Response and Prognosis

Response to therapy was assessed using morphological analysis, flow cytometry, and genetic testing. CR was defined as <5% bone marrow blasts in morphology regardless of cell count recovery, negative MRD, and negative high-risk genotype if existing. Bone marrow aspiration detection was performed every month for half a year and every 3 months in 2 years after CAR-T therapy. Relapsed disease was defined as the reappearance of blasts in the blood or bone marrow or in an extramedullary site after CR. Overall survival (OS) was defined as the time from infusion to the date of death or the last follow-up. Progression-free survival (PFS) was calculated from the date of CR to the date of relapse, death, or the last follow-up.

### Collection of Clinical Laboratories

Peripheral blood was collected for concentrations of inflammatory factors. The baselines of inflammatory factors were defined as concentrations at day 0 when CAR-T cell was infused. Peak levels were regarded as the maximum concentration before the administration of Tocilizumab or 1 month after CAR-T infusion in patients receiving Tocilizumab or not, respectively. Fold change was defined as the ratio of peak to baseline. Inflammatory factors that changed significantly during the process of CAR-T therapy included interleukin (IL)-2, IL-4, IL-6, IL-10, tumor necrosis factor alpha (TNF-α), interferon gamma (IFN-γ), C-reactive protein (CRP), and ferritin (FER), which were evaluated in the study.

### Statistical Analyses

All measurement data were described using median and range and compared using Mann–Whitney tests. Enumeration data were presented as frequency (%) and compared using chi-square tests or Fisher’s exact test. Follow-up time, OS, and LFS were estimated using the Kaplan–Meier method, whereas differences between groups were evaluated using log-rank test. Cutoffs were calculated by establishing ROC curves. All tests were two-sided, and p *<* 0.05 was considered statistically significant. Data were analyzed and presented using GraphPad Prism version 9.

## Results

### Patient Characteristics and Management Post-infusion

From May 2018 to September 2021, a total of 75 patients with R/R B-ALL in our phrase I/II clinical trials received CAR-T cells. Excluding five patients receiving secondary infusion after murine CAR-T therapy and three patients losing examination data, 67 patients were enrolled in our retrospective study. Baseline characteristics of all 67 patients and the subgroup analysis of the toci and non-toci groups were summarized (**Table 1**). As shown, two groups were similar with regard to normal demographics and previous therapies. However, tumor burdens were higher in the toci group with significant statistical difference.

**Table 1:** Characteristics and treatment of patients and comparison of subgroups.

CHARACTERISTIC	Total (N = 67)	Toci (N = 32)	Non-toci (N = 35)	p
Median age (range), years	29 (13–65)	28 (14–58)	30 (18–65)	0.918
Male, No. (%)	35 (52.2)	13 (40.6)	22 (62.9)	0.068
Previous therapies				
Median lines of therapy (range)	3 (1–10)	3 (1–10)	3 (1–10)	0.088
Allogeneic SCT, No (%)	12 (18.9)	7 (21.9)	5 (14.3)	0.418
Primary refractory disease, No (%)	29 (43.3)	12 (37.5)	17 (48.5)	0.361
Baseline disease burden				
Bone marrow blasts, %				
>50	14 (20.9)	12 (37.5)	2 (5.7)	
25–50	13 (19.4)	8 (25)	5 (14.3)	
5–25	14 (20.9)	4 (12.5)	10 (28.6)	
<5	26 (38.8)	8 (25)	18 (51.4)	
Median blasts, range	16.53 (0–95.18)	30.60 (0–95.18)	4.83 (0–90)	0.001
High-risk phenotype or genotypes	23 (34.3)	8 (25)	15 (42.9)	0.124
Infusion of humanized CAR-T, No (%)	34 (50.7)	19 (59.4)	15 (42.9)	0.177
Doses of CAR-T, range (*10^6^/kg)	4 (2-6)	4 (2-4)	4 (2-6)	>0.999
Corticosteroids, No (%)	23 (34.3)	17 (53.1)	6 (17.4)	0.002

SCT, stem cell transplantation.

Patients receiving murine or humanized CD19 CAR-T cells accounted for 49.3% and 50.3%, respectively. After infusion, CRS occurred in 43 patients with 27 patients in grade 1, 11 patients in grade 2, 4 patients in grade 3, 1 patient in grade 4, and 1 patient in grade 5. The median onset and duration of CRS was 6 days (range, 2–11) and 5 days (range, 2–21), respectively. Tocilizumab was administrated in 33 patients including 17 patients with grade 1, 9 patients with grade 2, 5 patients with grade 3–5 CRS, and one patient with headache and elevated IL-6 in peripheral blood and cerebrospinal fluid instead of symptoms of CRS. One to four doses of Tocilizumab were used to suppress CRS with median dose of 480 mg (range, 160–800). The dosing interval of tocilizumab was 24–48 h. Corticosteroids were used in 23 patients, and the cumulative dexamethasone-equivalent corticosteroid dose was 62.5 mg (range, 7.5–302.5). Continuous dosing of corticosteroids was administrated until the patients’ symptoms improved. Significantly higher usage of corticosteroids appeared in the toci group. Of the total 67 patients, 86.6% (N=58) responded and 85% (N=57) achieved complete remission with MRD and genotype negativity.

### Usage of Tocilizumab Can Be Advanced to Grade 2 CRS

**Figure 1:**
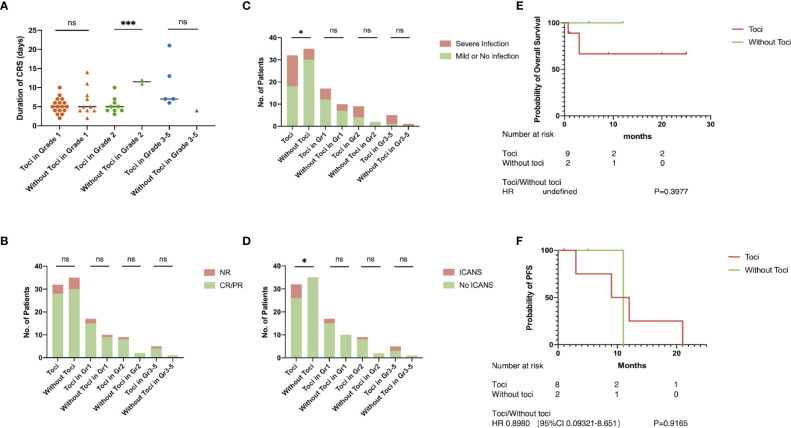
Tocilizumab shortened the duration of CRS. The grading of CRS and whether to use Tocilizumab were the basis for grouping. **(A)** Duration of CRS; **(B)** objective response rate; **(C)** incidence of severe infection; **(D)** incidence of ICANS; **(E)** overall survival rate in patients with grade 2 CRS; **(F)** progression-free survival in patients with grade 2 CRS. *p < 0.05; ***p < 0.001.

Patients’ clinical symptoms including pyrexia, hypotension, and hypoxemia were the main basis for CRS grading according to ASTCT criteria. The toci and non-toci groups had similar duration of CRS in grade 1, grade 3–5, and total patients. However, in patients developing grade 2 CRS, those treated with Tocilizumab had significantly shorter duration of CRS than those without Tocilizumab (p=0.0004, **Figure 1A**). Although the non-toci group experienced less severe infections or ICANS when comparing to the toci group, Tocilizumab did not amplify the incidence of these adverse events in the subgroup grade 2 (p=0.4545, **Figure 1C**; p>0.9999, **Figure 1D**). There was no difference between the toci and non-toci groups in terms of complete remission rate at 1 month (**Figure 1B**) and long-term efficacy including overall survival (grade 1, p=0.1893; grade 3–5, p=0.6547, **Supplementary Figure 2**; grade 2, p=0.3977, **Figure 1E**) and progression-free survival (grade 1, p=0.1309; grade 3–5, p=0.2743, **Supplementary Figure 2**; grade 2, p=0.9186, **Figure 1F**) regardless of CRS grading.

### Two Patients Progressed to Severe CRS After Early Treatment of Tocilizumab

Patient A received CAR-T cells on September 2020 and had a fever with hypoxemia on day 5 post-infusion, which was defined as grade 2 CRS. He was given Tocilizumab twice on days 7 and 9 when the conventional antipyretic therapy was ineffective. However, the condition progressed rapidly right after the second infusion of Tocilizumab and respiratory support system, and vasopressors were required because CRS had upgraded to grade 4. After effective application of corticosteroids and supportive therapies, the patient gradually recovered and achieved complete remission within 21 days.

**Figure 2:**
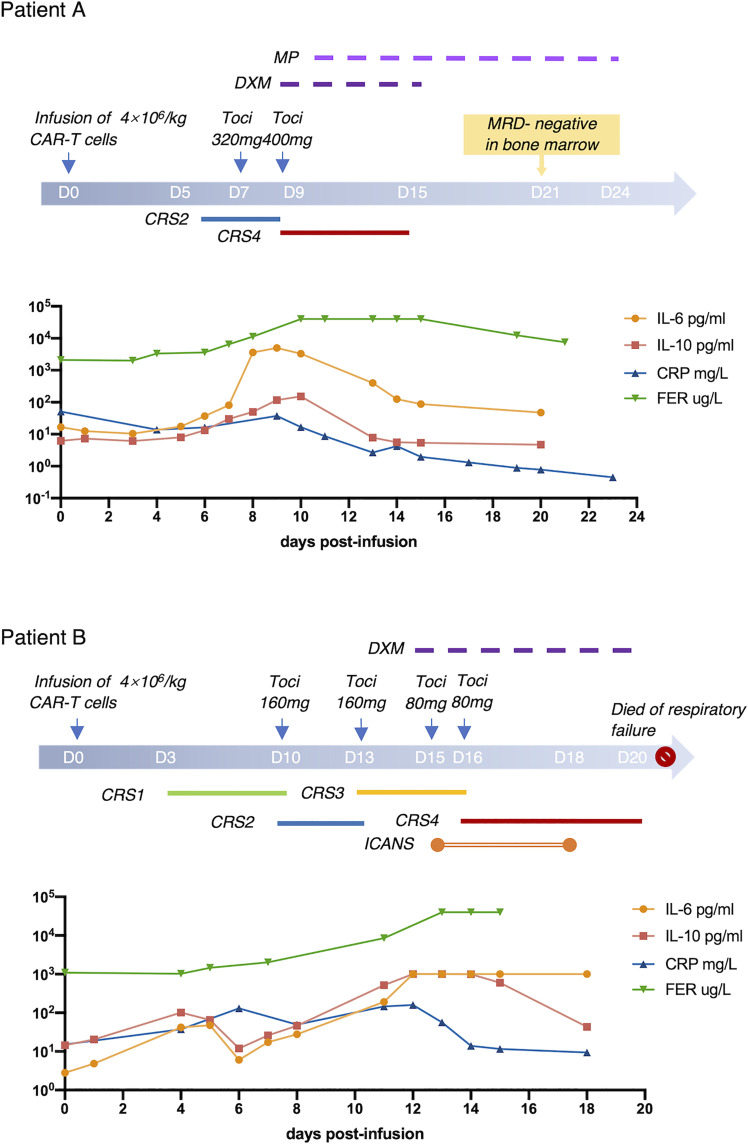
Two Cases With Severe CRS. Progression and treatment of two typical patients with severe CRS. MP, methylprednisolone; DXM, dexamethasone.

Patient B who received CAR-T cells on September 2018 seemed to have similar progression of CRS. She experienced prolonged mild CRS during days 3–13. Tocilizumab had been administrated twice when the patient was in grade 2 CRS. However, the intervention did not restrain the progression of CRS, and unfortunately, the patient died of respiratory failure related to severe CRS on day 20 (**Figure 2**).

### Cytokines Related to CRS and the Cutoffs

Laboratory biomarkers of inflammation were evaluated in all treated subjects from the time of CAR-T cell infusion to 1 month after infusion. There was no difference between cytokine and inflammation indicator levels of patients before or after treatment of Tocilizumab except for IL-6 (**Supplementary Figure 3**). Thus, Tocilizumab could increase the level of IL-6 in peripheral blood exponentially; we defined peak level of IL-6 in the toci group as the maximum before administration of Tocilizumab.

**Figure 3:**
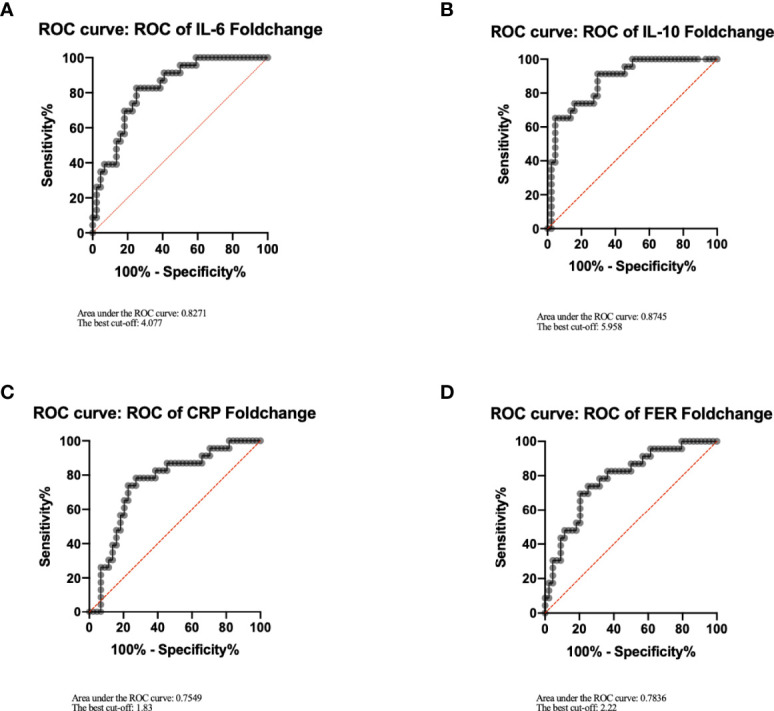
Cutoffs of IL-6, IL-10, CRP, FER. IL-6 **(A)**, IL-10 **(B)**, CRP **(C)**, FER **(D)** were analyzed by ROC curve using GraphPad Prism, and the best cutoffs were determined according to the coordinates of the ROC curve. Youden index=sensitivity+specificity−1. Its maximum value corresponds to the optimal cutoff.

Both peak amount and fold change of IL-6, IL-10, CRP, and FER had significant statistical differences between patients with or without CRS (**Supplementary Figure 4**), which indicated that these laboratory markers presented a positive correlation with the occurrence of CRS. Therefore, we grouped the patients into low and high levels of IL-6, IL-10, CRP, or FRE, respectively, on the basis of the cutoffs of ROC curve (**Figure 3**).

### Fold Change of IL-6 and CRP Back the Use of Tocilizumab

**Figure 4:**
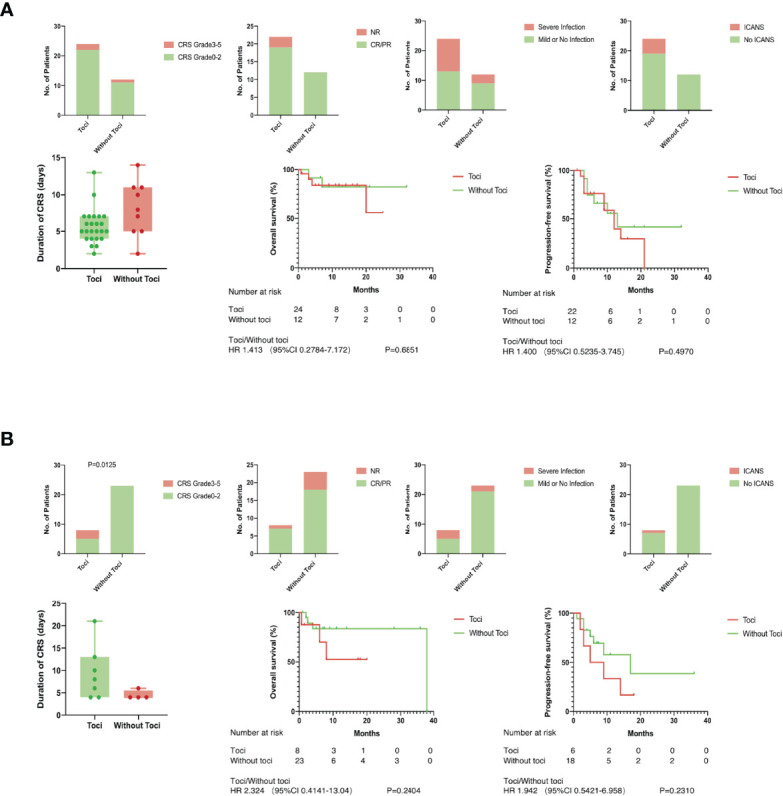
Tocilizumab induced severe CRS in low level group. There was no significantly difference between patients with toci and without toci except for one. **(A)** Duration of CRS, incidence of severe infection and ICANS, objective response rate in short-term, overall survival and progression-free survival in long term were compared between patients with or without toci in high level group of IL-6; **(B)** Duration of CRS, incidence of severe infection and ICANS, objective response rate in short-term, overall survival, and progression-free survival in the long term were compared between patients with or without toci in low level group of IL-6.

The efficacy of Tocilizumab on CRS, the influence on other adverse events, and the effect on short- or long-term anti-leukemia capacity were evaluated in high- and low-level groups, respectively. In patients with less than fourfold increase in IL-6, severe CRS was more likely to take place in patients treated with Tocilizumab (37.5% versus 0%, **Figure 4B**). A shorter duration of CRS could be observed when treatment was given at a remarkably elevated IL-6 (median, 5; range, 3–13 days versus median, 8; range, 2–14 days, p=0.0575, **Figure 4A**), but without statistical difference. When patients were divided according to the level of CRP, it was worth noting that patients with elevated CRP had an additional chance of developing serious infections after receiving Tocilizumab (47.8% versus 11.8%, p=0.0204, **Supplementary Figure 6A**). There was no significantly difference between the two groups parted by IL-10 or FER in all aspects (**Supplementary Figures 7, 8**).

## Discussion

CAR-T cell therapy, as a promising novel immunotherapy, resulted in unprecedented remission rates in patients with B-ALL. However, frequent, even potentially life-threatening adverse events represented by CRS limited patients’ survival and quality of life after treatment. By binding to IL-6R, Tocilizumab accurately inhibits the downward transmission of IL-6 signaling pathways and effectively reduces the secretion of various acute inflammatory proteins such as CRP, and is considered to be a safe drug that suppresses CRS without affecting CAR-T cell expansion and long-term remission (2, 4, 19). The US Food and Drug Administration had approved Tocilizumab for the treatment of severe CRS, but several studies (15, 16) showed that preemptive usage of Tocilizumab might facilitate the better control of CRS. NCCN (8) had also formulated different timings for infusing Tocilizumab in each commercial CAR-T products, especially the immediate administration of Tocilizumab when CRS occurred within 72 h after infusion of axicabtagene ciloleucel or brexucabtagene autoleucel regardless of grading. However, the ideal timing of the use of Tocilizumab that is universal to different kinds of CAR-T cells has not yet been determined.

Before discussing the effect of tocilizumab on CRS, the influence of other factors on the development of CRS should be excluded. The state of T cells used to manufacture CAR-T cells can affect the proliferation and cytotoxicity of CAR-T cells *in vivo* and, therefore, contributes to CRS. The state of T cells is determined by the baseline characteristics of patients including age, disease, chronic infections, tumor burden, and previous treatments, especially SCT (20). In our study, all participants were patients with ALL, and their baseline conditions are presented in **Table 1** and analyzed between subgroups. Patients in the toci group had a significantly higher tumor load, which would be discussed later, but there was no statistic difference in other aspects.

On the basis of clinical symptoms, the ASTCT grading system proved to be easily applicable, and its accuracy was confirmed in the incidence of adverse reactions in previous clinical trials (7). These clinical symptoms, including fever, hypotension, and hypoxemia, can be used as factors in determining whether to take interventions or not. In our clinical trials, patients who developed grade 2 CRS and received Tocilizumab terminated inflammation responses earlier than those without Tocilizumab, revealing that patients with persistent fever and mild hypotension or hypoxemia should be considered for Tocilizumab intervention. In the whole, the use of Tocilizumab made a statistically significant difference in the occurrence of adverse events such as ICANS and severe infection. In our study, all patients who developed ICANS had used Tocilizumab, but 50% (n=3) of the patients had neurological symptoms such as impaired consciousness before intervention. Besides, the incidence of ICANS in patients using Tocilizumab was not significantly increased in each grade of CRS. To our knowledge, no previous studies have found a relationship among CRS, Tocilizumab, and severe infection. It could cause a mild reduction in neutrophil count among healthy people (14) and increased susceptibility to bacterial infections in patients with rheumatologic diseases. However, when it comes to CAR-T therapy, the use of Tocilizumab is unlikely to be a direct element in infection due to multiple factors such as the lymphodepleting chemotherapy and hematological toxicity of the CAR-T cells. Similarly, the incidence of severe infections did not increase in patients receiving Tocilizumab in each grade of CRS.

Statistically, the use of Tocilizumab at grade 2 CRS can effectively inhibit the continuous progression of CRS without affecting the efficacy and safety of CAR-T therapies. However, the progression and treatment process of two patients provided us with a new orientation for evaluating intervention. Both of these two patients received Tocilizumab at grade 2 CRS and progressed to grade 4 with mildly elevated IL-6 before treatment of Tocilizumab. A study had proven that elevated levels of IL-6 could predict the use of invasive mechanical ventilation in patients with severe COVID-19 after administration of Tocilizumab (21). Analysis of two cases and the previous study have demonstrated that apart from clinical symptoms, laboratory biomarkers, especially IL-6, may also be able to guide medications.

Given the large differences in baseline levels among patients, we considered that laboratory biomarkers of which both the absolute and the fold change made statistical differences can be included in the following discussion. Patients with IL-6 elevating below four times are more likely to develop severe CRS after receiving Tocilizumab. This phenomenon can be caused by a variety of factors, including precise antagonism of Tocilizumab to IL-6 and delayed treatment such as corticosteroids or plasmapheresis. The blockade of IL-6 pathway would effectively reduce the production of CRP and control the patients temperature. However, at the same time, it may conceal the apparent signs of the ongoing inflammatory storm inside the patients, and other treatments could not be applied in a timely manner. In the group with remarkable elevated IL-6, the use of Tocilizumab did not significantly inhibit the development of CRS and cause changes in safety and efficacy, which is inconsistent with our hypothesis. It is widely acknowledged that the occurrence of inflammation is positively correlated with the tumor burden before treatment. Thus, it is reasonable to speculate that the inconspicuous inhibition of Tocilizumab in CRS may be due to a significant difference in tumor burden between the toci and non-toci groups.

Due to the block of receptors using Tocilizumab, dissociative IL-6 in the peripheral blood rose rapidly. It has been reported that cytokines accelerated the activation of endothelial cells, which results in the injury of blood–brain barrier (22, 23). An experiment *in vivo* had proved that IL-6 contributed to blood–brain barrier dysfunction *via* JAK-STAT signaling pathway in tumor microenvironment (24). On the one hand, significantly increased pro-inflammation cytokines diffused to the central nervous system. On the other hand, impaired blood–brain barrier could not inhibit the entry of CAR-T cells, which might targeted mural cells expressing CD19 (25). Both sides contributed to the occurrence of ICANS. Several studies (17, 26, 27) disclosed that Tocilizumab given prophylactically would not benefit or even increase the rates of neurotoxicity. In our study, there was no significant difference between two groups in the incidence of ICANS when the level of IL-6 lifted over four times. If we had a sufficient number of ICANS, it would be reasonable to set up a maximum level of IL-6 beyond which measures were demanded to prevent ICNAS after using Tocilizumab. Severe CRS has been demonstrated to be associated with infections (28, 29) which may be related to liver secreting acute inflammatory proteins induced by IL-6 and pancytopenia caused by severe CRS (28). In our study, patients with elevated CRP over 1.83 times were more likely to develop severe infections after receiving Tocilizumab, which suggested that we should pay more attention to the patients’ infection progression after administration of Tocilizumab and intervene as soon as possible.

**Figure 5:**
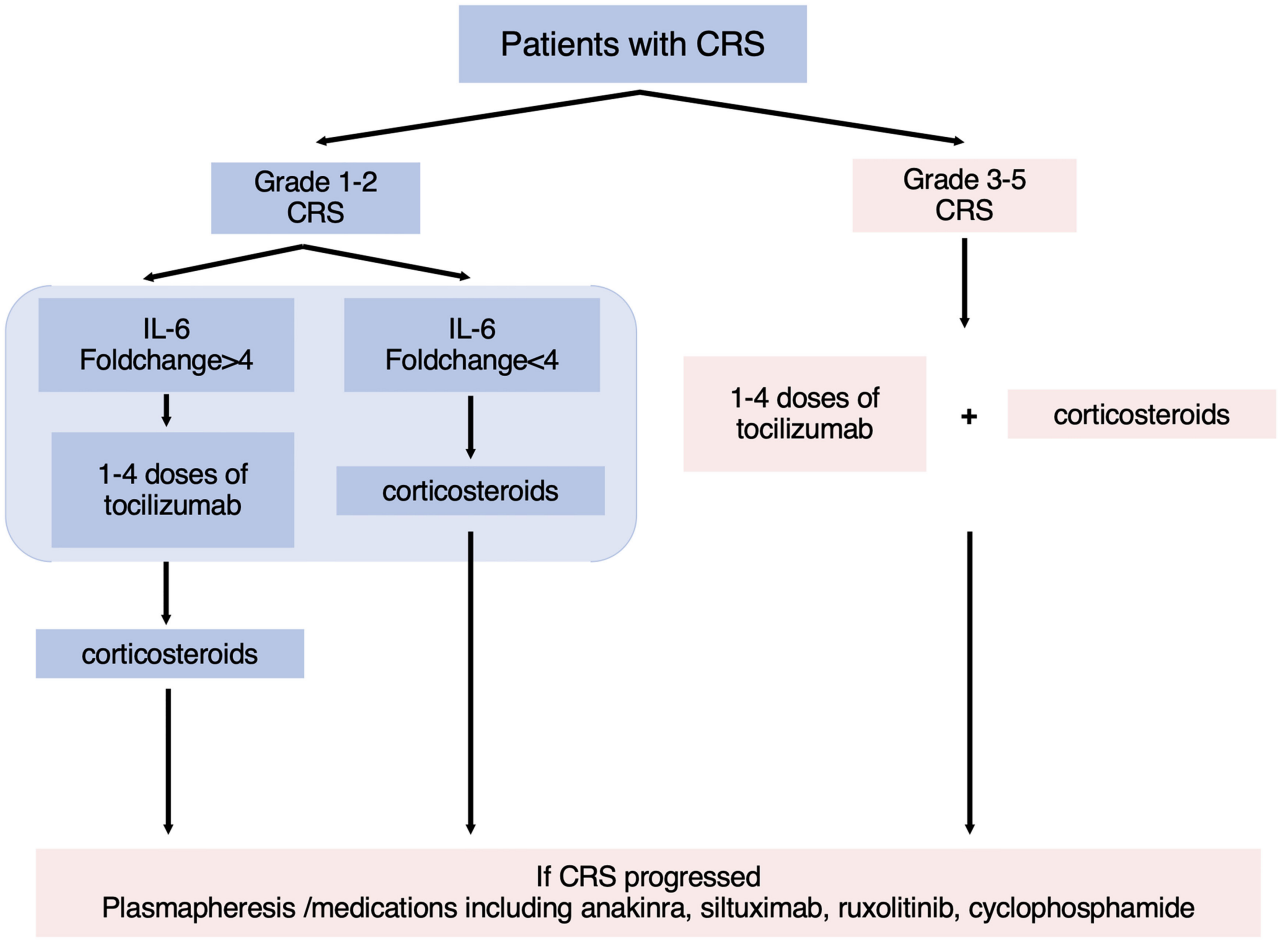
The flowchart of guidelines for the treatment of CRS. According to the ASTCT grading system and level of IL-6, patients were recommended for different treatment options.

In summary, we validated the guiding significance of the grading system for the early application of Tocilizumab while introducing level of IL-6 to consummate individualized treatment for patients with different inflammatory responses. Through a retrospective study of 67 patients with B-ALL in our center, Tocilizumab was recommended for patients whose clinical symptoms had meet the standard of ASTCT grade 2 and whose concentration of IL-6 had increased by more than four times (**Figure 5**). For patients whose clinical symptoms had up to standard with IL-6 rising inconspicuously, they were recommended to receive corticosteroids in advance to inhibit the progression of CRS. For patients with CRP increasing >1.83 times, signs and indicators of infection should be strictly monitored after treatment of Tocilizumab to avoid the occurrence of fatal infection. This study still has some limitations. Due to the relatively aggressive treatment regimen for CRS in our center, few patients did not use tocilizumab after the onset of CRS. Although based on the available data, we presented a statistical difference in the duration of CRS between the two groups in grade 2 CRS, a larger sample size is needed to further validate this conclusion. Moreover, 15% of patients lost contact 1 month after CAR-T therapies so the follow-ups were terminated. Nonetheless, our conclusion can guide clinical medication to some extent and improve the safety of CAR-T cell infusion.

## Data Availability Statement

The original contributions presented in the study are included in the article/**Supplementary Material**. Further inquiries can be directed to the corresponding author/s.

## Ethics Statement

The studies involving human participants were reviewed and approved by the Medical Ethics Committee of Union Hospital, Tongji Medical College, Huazhong University of Science and Technology. Written informed consent to participate in this study was provided by the participants’ legal guardian/next of kin.

## Author Contributions

HM designed the study. YL, MD, YZ, and FZ contributed to clinical data collection and analysis. YZ, ZW, and FZ wrote the first version of the manuscript. CLi, MD, WL, FZ, and HM revised the manuscript. HK, CLu, and HM performed the clinical trial and provided patient care. All authors read the final manuscript and have agreed to be co-authors.

## Funding

This work was supported by the National Key Research and Development Program of China (No. 2019YFC1316203), National Natural Science Foundation of China (No. 82070124) and Natural Science Foundation of Hubei Province (No. 2020CFA065).

## Conflict of Interest

The authors declare that the research was conducted in the absence of any commercial or financial relationships that could be construed as a potential conflict of interest.

The reviewer CL declared a shared parent affiliation with the authors to the handling editor at the time of review.

## Publisher’s Note

All claims expressed in this article are solely those of the authors and do not necessarily represent those of their affiliated organizations, or those of the publisher, the editors and the reviewers. Any product that may be evaluated in this article, or claim that may be made by its manufacturer, is not guaranteed or endorsed by the publisher.
