# Supplementary material for: Timing of Tocilizumab Administration Under the Guidance of IL-6 in CAR-T Therapy for R/R Acute Lymphoblastic Leukemia
Source: Front Immunol. 2022 Jun 21;13:914959. doi: 10.3389/fimmu.2022.914959 (PMC9253384; doi:10.3389/fimmu.2022.914959)
Supplement: Supplementary file 1 [file DataSheet_1.pdf]

Supplement Materials

Supplement Figure 1 An inclusion flow chart of our two clinical trials.

Supplement Figure 2 Overall survival and progression-free survival of patients in different grade of CRS (A) and of the toci and without toci groups in the total (B), grade 1 CRS (C), grade 2 (D) and grade3-5 (E) CRS of patients. There is no significantly difference among all groups.

Supplement Figure 3 Peaks level of IL-6, IL-10, IL-2, IL-4, TNF-α, IFN-γ, CRP and FER before and after the use of tocilizumab

Supplement Figure 4 Biomarkers related to CRS
Peak concentration and foldchange of cytokines and inflammatory factors were evaluated between patients with or without CRS. The peak concentration of IL-6 in patients receiving Tocilizumab was defined as the maximum before treatment. The peak concentration of IL-6 (C) in patients without Tocilizumab and IL-2 (A), IL-4 (B), IL-10 (D), TNF-α (E), IFN-γ (F), CRP (G), FER (H) in all patients was defined as the maximum in one month after infusion.

Supplement Figure 5 Kinetics of biomarkers related to CRS
Dynamic changes of IL-2, IL-4, IL-6, IL-10, TNF-α, IFN-γ, CRP, FER in all patients with or without CRS. A significant increase of IL-6 and IL-10 was observed in CRS group.

Supplement Figure 6 Tocilizumab induced severe infection in high level group of CRP. There was no significantly difference between patients with toci and without toci except for one. (A) Duration of CRS, incidence of severe infection and ICANS, objective response rate in short-term, overall survival and progression-free survival in long term were compared between patients with or without toci in high level group of CRP; (B) Duration of CRS, incidence of severe infection and ICANS, objective response rate in short-term, overall survival and progression-free survival in long term were compared between patients with or without toci in low level group of CRP

Supplement Figure 7 There was no significantly difference between patients with toci and without toci. (A) Duration of CRS, incidence of severe infection and ICANS, objective response rate in short-term, overall survival and progression-free survival in long term were compared between patients with or without toci in high level group of IL-10; (B) Duration of CRS, incidence of severe infection and ICANS, objective response rate in short-term, overall survival and progression-free survival in long term were compared between patients with or without toci in low level group of IL-10

Supplement Figure 8 There was no significantly difference between patients with toci and without toci. (A) Duration of CRS, incidence of severe infection and ICANS, objective response rate in short-term, overall survival and progression-free survival in long term were compared between patients with or without toci in high level group of FER; (B) Duration of

CRS, incidence of severe infection and ICANS, objective response rate in short-term, overall survival and progression-free survival in long term were compared between patients with or without toci in low level group of FER

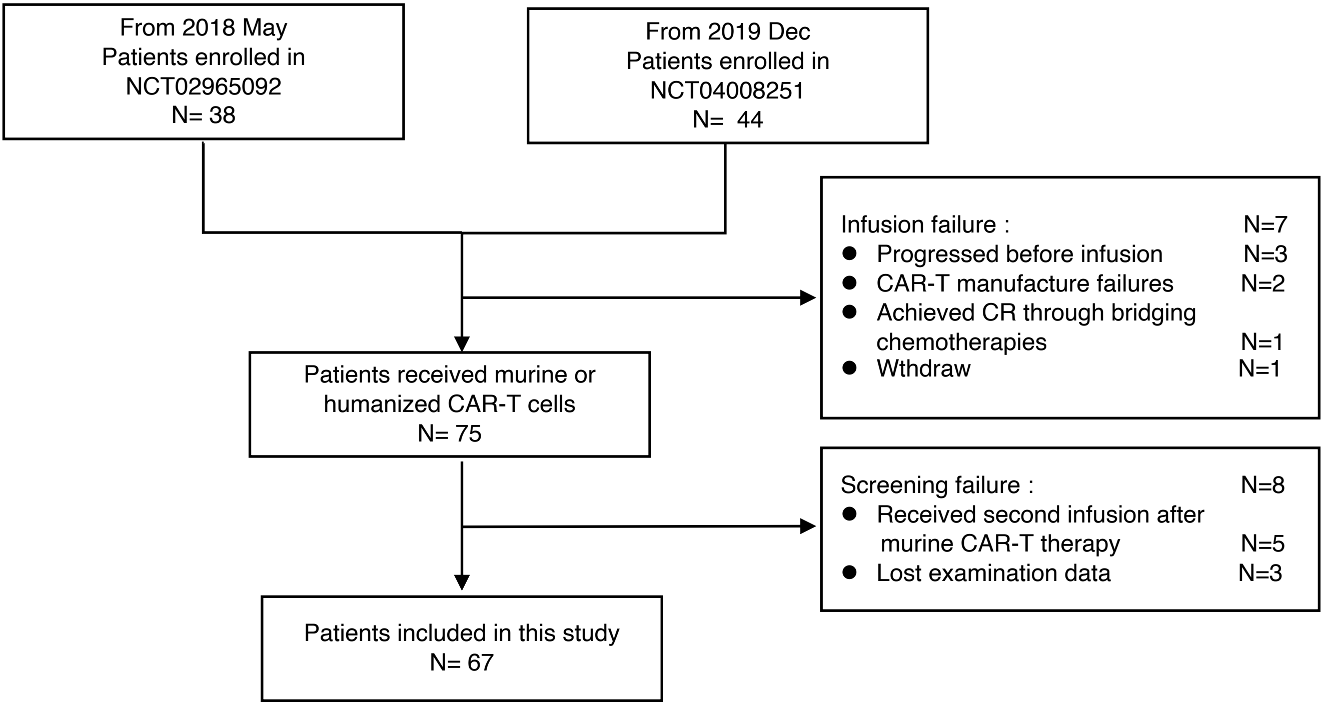
From 2018 May
Patients enrolled in
NCT02965092
N= 38
From 2019 Dec
Patients enrolled in
NCT04008251
N= 44
Infusion failure : N=7
Progressed before infusion N=3
CAR-T manufacture failures N=2
Achieved CR through bridging chemotherapies N=1
Wthdraw N=1
Patients received murine or humanized CAR-T cells
N= 75
Screening failure : N=8
Received second infusion after murine CAR-T therapy N=5
Lost examination data N=3
Patients included in this study
N= 67

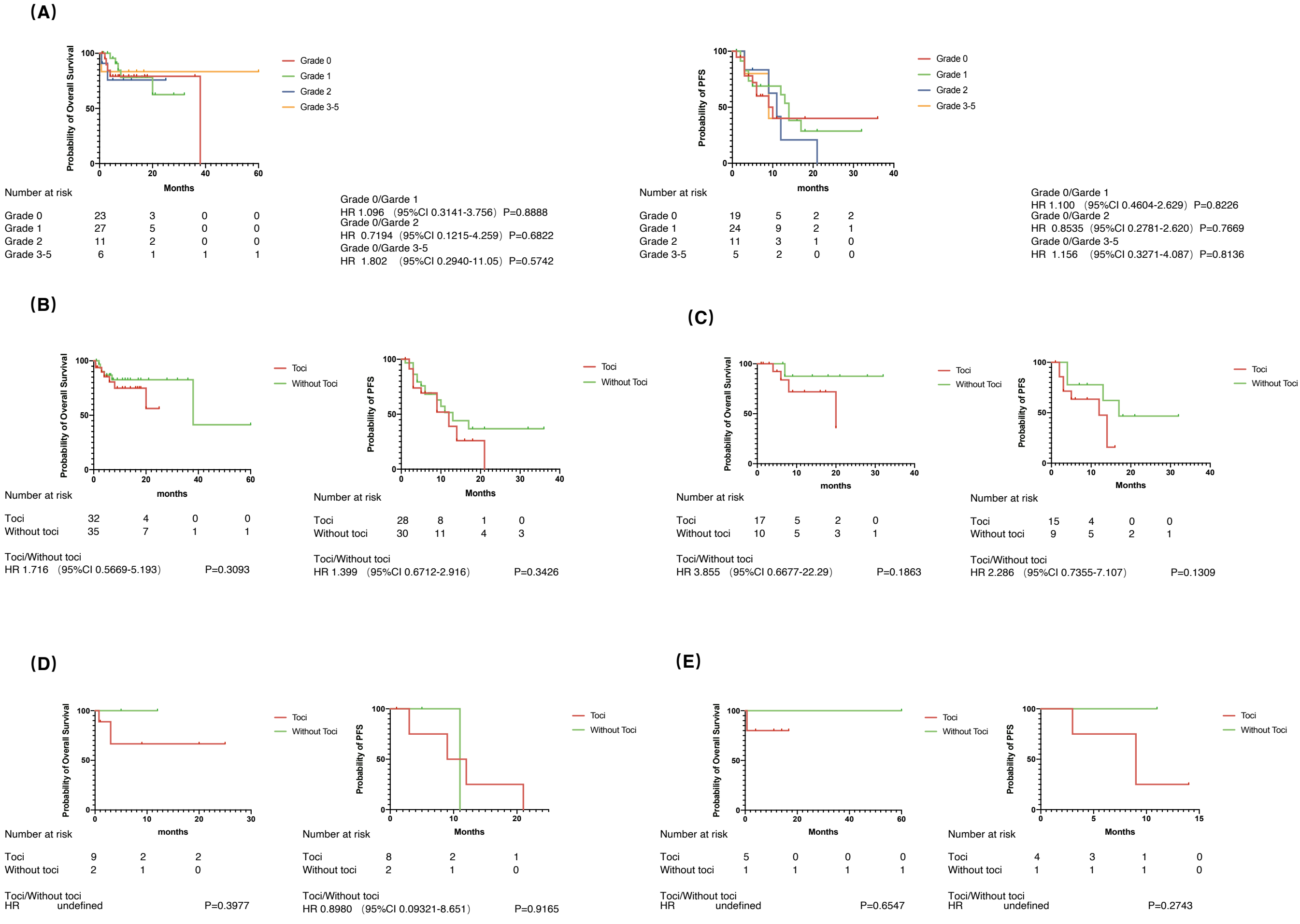
(A)
Probability of Overall Survival
Months
Grade 0
Grade 1
Grade 2
Grade 3-5
Number at risk
Grade 0 23 3 0 0
Grade 1 27 5 0 0
Grade 2 11 2 0 0
Grade 3-5 6 1 1 1
Grade 0/Garde 1
HR 1.096 (95%CI 0.3141-3.756) P=0.8888
Grade 0/Garde 2
HR 0.7194 (95%CI 0.1215-4.259) P=0.6822
Grade 0/Garde 3-5
HR 1.802 (95%CI 0.2940-11.05) P=0.5742
Probability of PFS
months
Grade 0
Grade 1
Grade 2
Grade 3-5
Number at risk
Grade 0 19 5 2 2
Grade 1 24 9 2 1
Grade 2 11 3 1 0
Grade 3-5 5 2 0 0
Grade 0/Garde 1
HR 1.100 (95%CI 0.4604-2.629) P=0.8226
Grade 0/Garde 2
HR 0.8535 (95%CI 0.2781-2.620) P=0.7669
Grade 0/Garde 3-5
HR 1.156 (95%CI 0.3271-4.087) P=0.8136
(B)
Probability of Overall Survival
months
Toci
Without Toci
Number at risk
Toci 32 4 0 0
Without toci 35 7 1 1
Toci/Without toci
HR 1.716 (95%CI 0.5669-5.193) P=0.3093
Probability of PFS
Months
Toci
Without Toci
Number at risk
Toci 28 8 1 0
Without toci 30 11 4 3
Toci/Without toci
HR 1.399 (95%CI 0.6712-2.916) P=0.3426
(C)
Probability of Overall Survival
months
Toci
Without Toci
Number at risk
Toci 17 5 2 0
Without toci 10 5 3 1
Toci/Without toci
HR 3.855 (95%CI 0.6677-22.29) P=0.1863
Probability of PFS
Months
Toci
Without Toci
Number at risk
Toci 15 4 0 0
Without toci 9 5 2 1
Toci/Without toci
HR 2.286 (95%CI 0.7355-7.107) P=0.1309
(D)
Probability of Overall Survival
months
Toci
Without Toci
Number at risk
Toci 9 2 2
Without toci 2 1 0
Toci/Without toci
HR undefined P=0.3977
Probability of PFS
Months
Toci
Without Toci
Number at risk
Toci 8 2 1
Without toci 2 1 0
Toci/Without toci
HR 0.8980 (95%CI 0.09321-8.651) P=0.9165
(E)
Probability of Overall Survival
Months
Toci
Without Toci
Number at risk
Toci 5 0 0 0
Without toci 1 1 1 1
Toci/Without toci
HR undefined P=0.6547
Probability of PFS
Months
Toci
Without Toci
Number at risk
Toci 4 3 1 0
Without toci 1 1 1 0
Toci/Without toci
HR undefined P=0.2743

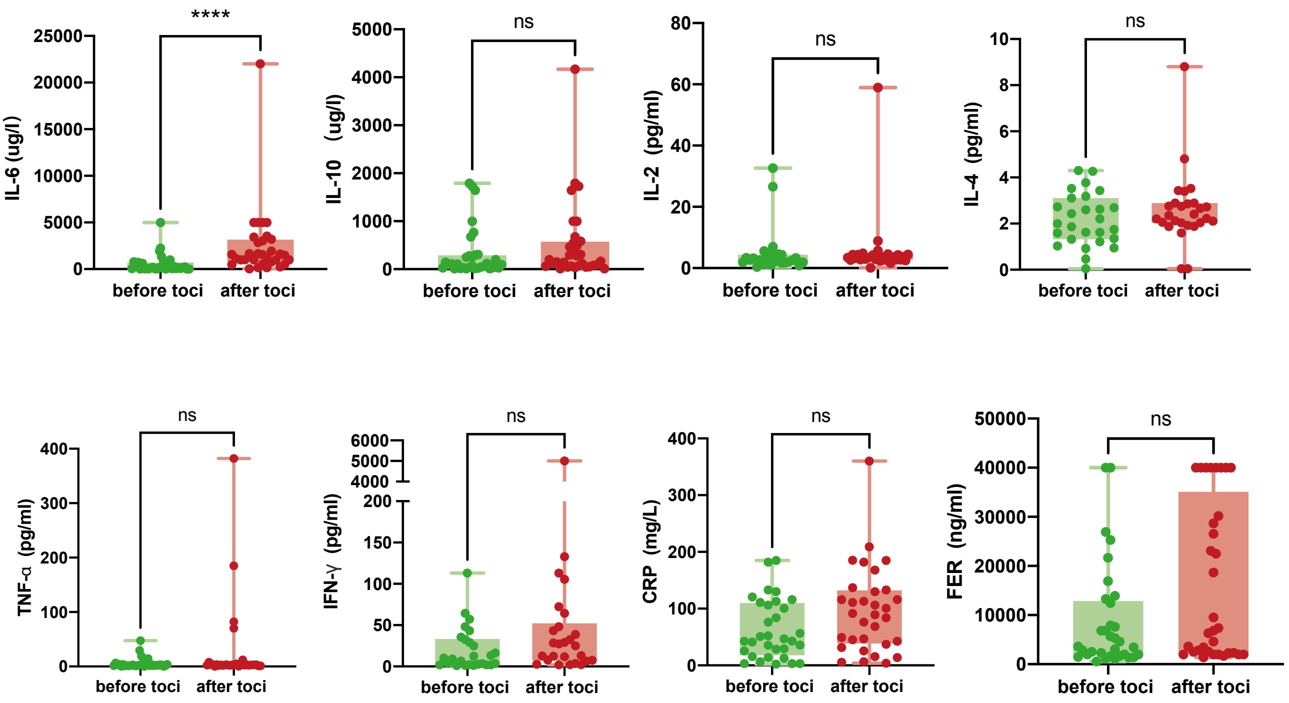

****
IL-6 (ug/l)
25000
20000
15000
10000
5000
0
before toci
after toci
ns
IL-10 (ug/l)
5000
4000
3000
2000
1000
0
before toci
after toci
ns
IL-2 (pg/ml)
80
60
40
20
0
before toci
after toci
ns
IL-4 (pg/ml)
10
8
6
4
2
0
before toci
after toci
ns
TNF-α (pg/ml)
400
300
200
100
0
before toci
after toci
ns
IFN-γ (pg/ml)
6000
5000
4000
200
150
100
50
0
before toci
after toci
ns
CRP (mg/L)
400
300
200
100
0
before toci
after toci
ns
FER (ng/ml)
50000
40000
30000
20000
10000
0
before toci
after toci

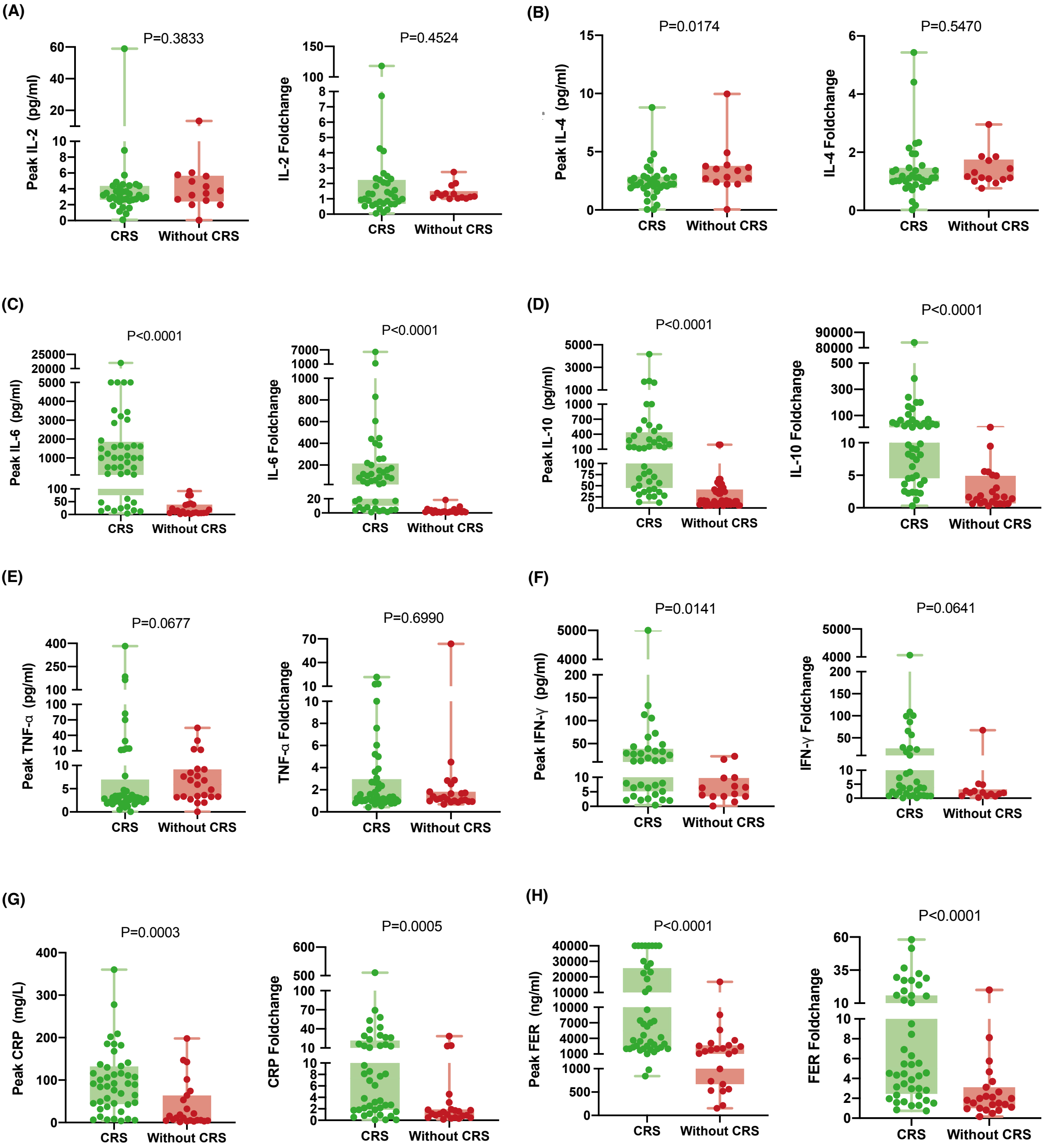

(A)
P=0.3833
Peak IL-2 (pg/ml)
P=0.4524
IL-2 Foldchange
(B)
P=0.0174
Peak IL-4 (pg/ml)
P=0.5470
IL-4 Foldchange
(C)
P<0.0001
Peak IL-6 (pg/ml)
P<0.0001
IL-6 Foldchange
(D)
P<0.0001
Peak IL-10 (pg/ml)
P<0.0001
IL-10 Foldchange
(E)
P=0.0677
Peak TNF-α (pg/ml)
P=0.6990
TNF-α Foldchange
(F)
P=0.0141
Peak IFN-γ (pg/ml)
P=0.0641
IFN-γ Foldchange
(G)
P=0.0003
Peak CRP (mg/L)
P=0.0005
CRP Foldchange
(H)
P<0.0001
Peak FER (ng/ml)
P<0.0001
FER Foldchange
CRS
Without CRS

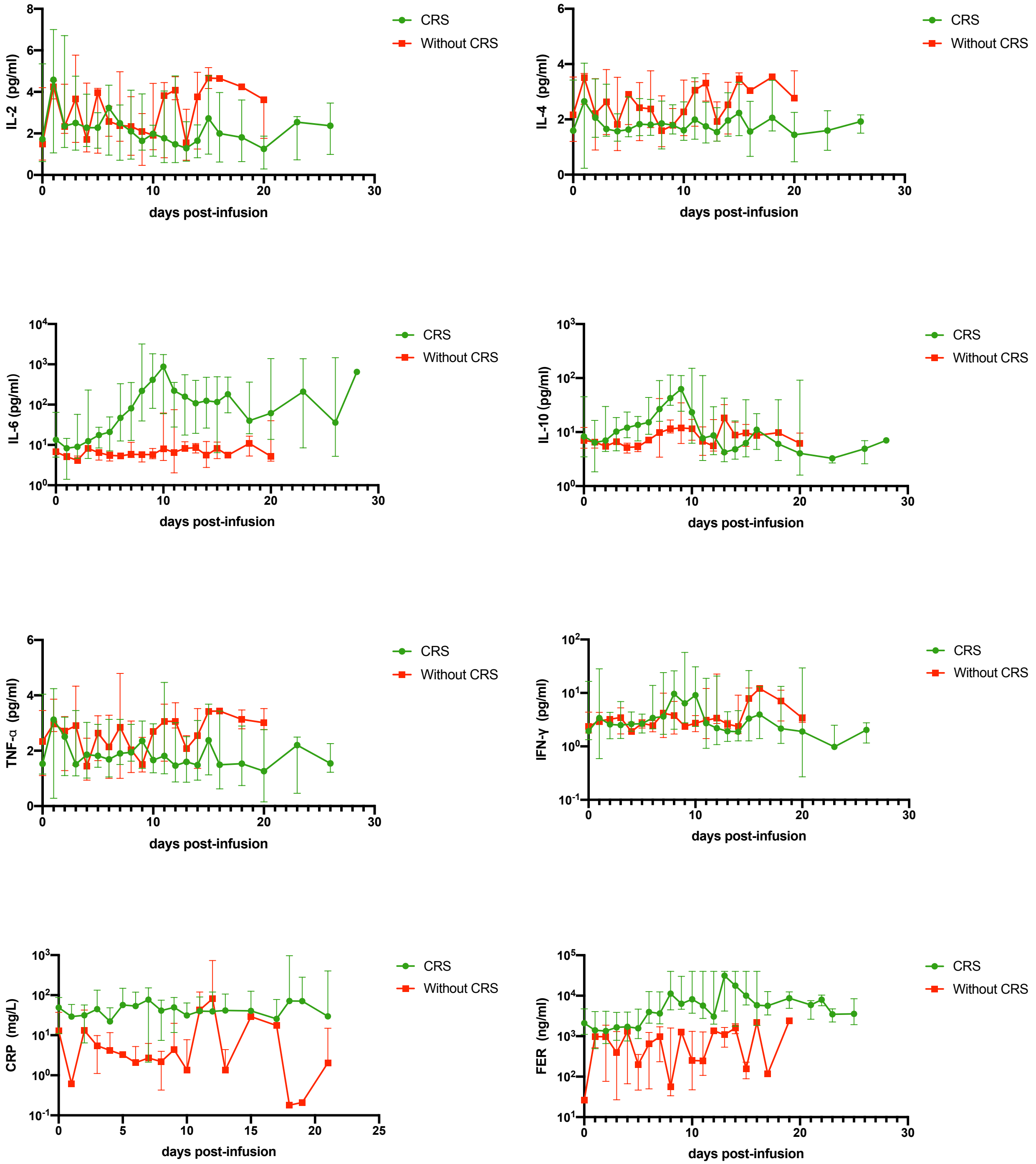

IL-2 (pg/ml)
CRS
Without CRS
days post-infusion
IL-4 (pg/ml)
CRS
Without CRS
days post-infusion
IL-6 (pg/ml)
CRS
Without CRS
days post-infusion
IL-10 (pg/ml)
CRS
Without CRS
days post-infusion
TNF-α (pg/ml)
CRS
Without CRS
days post-infusion
IFN-γ (pg/ml)
CRS
Without CRS
days post-infusion
CRP (mg/L)
CRS
Without CRS
days post-infusion
FER (ng/ml)
CRS
Without CRS
days post-infusion

(A)

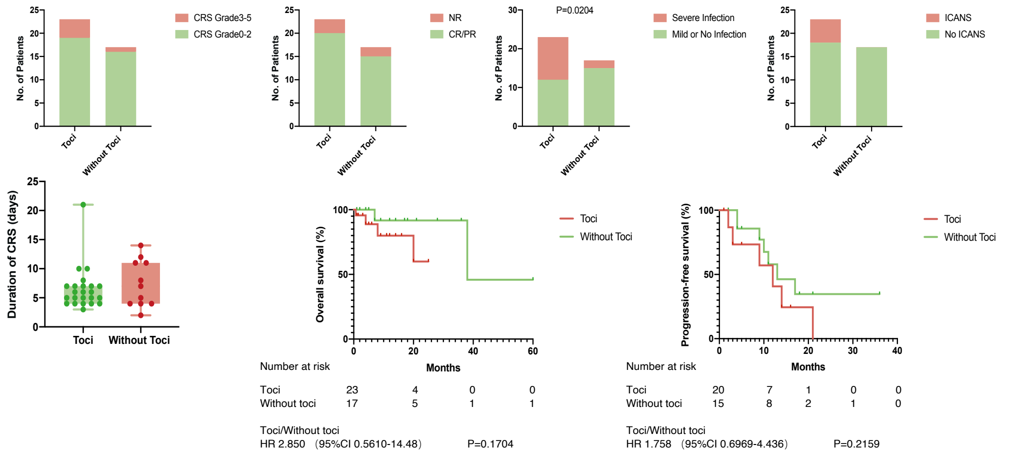

CRS Grade3-5
CRS Grade0-2
NR
CR/PR
P=0.0204
Severe Infection
Mild or No Infection
ICANS
No ICANS
No. of Patients
Toci
Without Toci
Duration of CRS (days)
Overall survival (%)
Progression-free survival (%)
Months
Number at risk
Toci 23 4 0 0
Without toci 17 5 1 1
Toci/Without toci
HR 2.850 （95%CI 0.5610-14.48） P=0.1704
Number at risk
Toci 20 7 1 0 0
Without toci 15 8 2 1 0
Toci/Without toci
HR 1.758 （95%CI 0.6969-4.436） P=0.2159

(B)

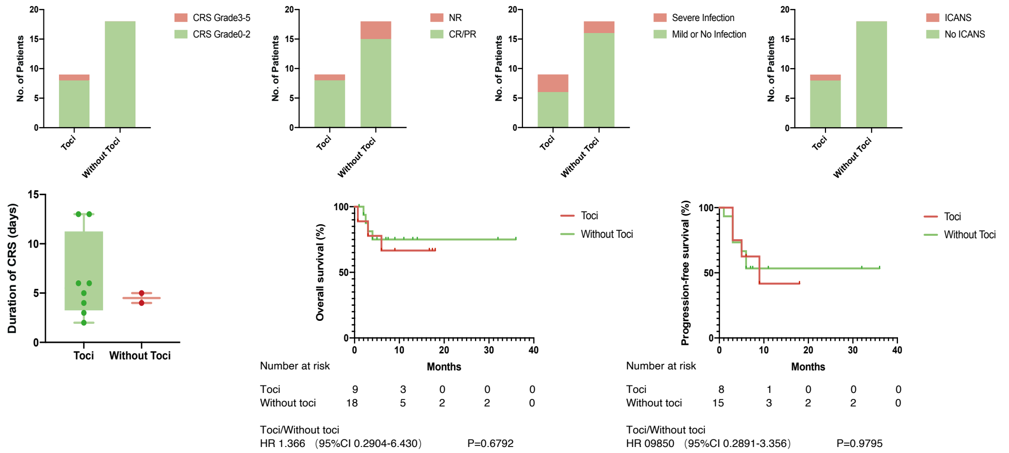

CRS Grade3-5
CRS Grade0-2
NR
CR/PR
Severe Infection
Mild or No Infection
ICANS
No ICANS
No. of Patients
Toci
Without Toci
Duration of CRS (days)
Overall survival (%)
Progression-free survival (%)
Months
Number at risk
Toci 9 3 0 0 0
Without toci 18 5 2 2 0
Toci/Without toci
HR 1.366 （95%CI 0.2904-6.430） P=0.6792
Number at risk
Toci 8 1 0 0 0
Without toci 15 3 2 2 0
Toci/Without toci
HR 09850 （95%CI 0.2891-3.356） P=0.9795

(A)

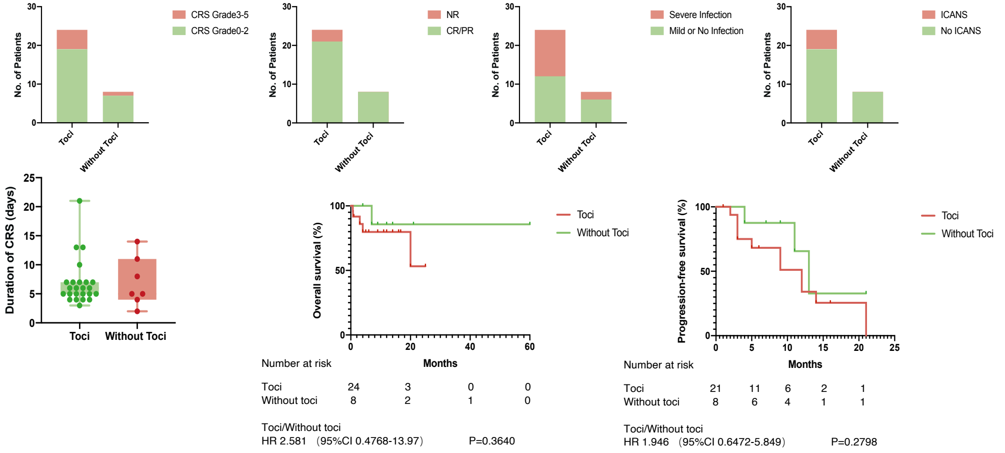

No. of Patients
CRS Grade3-5
CRS Grade0-2
NR
CR/PR
Severe Infection
Mild or No Infection
ICANS
No ICANS
Toci
Without Toci
Duration of CRS (days)
Overall survival (%)
Progression-free survival (%)
Months
Number at risk
Toci 24 3 0 0
Without toci 8 2 1 0
Toci/Without toci
HR 2.581 (95%CI 0.4768-13.97) P=0.3640
Toci 21 11 6 2 1
Without toci 8 6 4 1 1
Toci/Without toci
HR 1.946 (95%CI 0.6472-5.849) P=0.2798

(B)

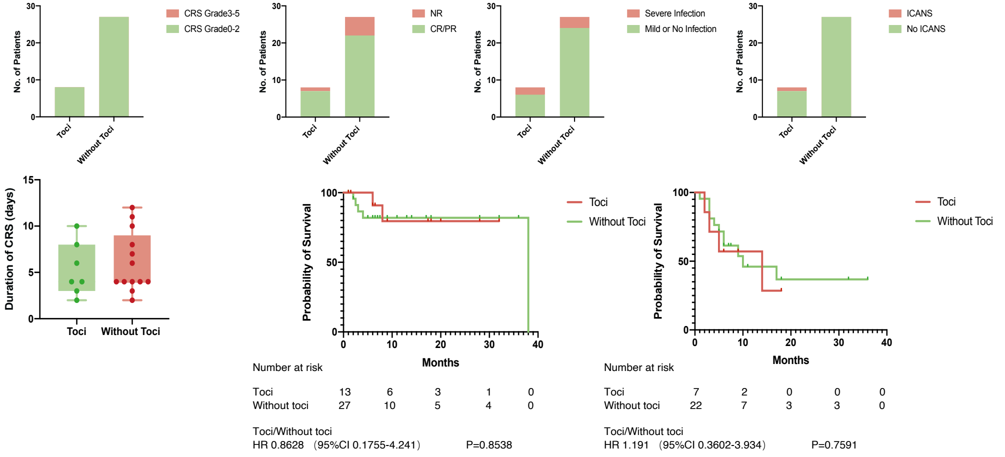

No. of Patients
CRS Grade3-5
CRS Grade0-2
NR
CR/PR
Severe Infection
Mild or No Infection
ICANS
No ICANS
Toci
Without Toci
Duration of CRS (days)
Probability of Survival
Months
Number at risk
Toci 13 6 3 1 0
Without toci 27 10 5 4 0
Toci/Without toci
HR 0.8628 (95%CI 0.1755-4.241) P=0.8538
Toci 7 2 0 0 0
Without toci 22 7 3 3 0
Toci/Without toci
HR 1.191 (95%CI 0.3602-3.934) P=0.7591

(A)

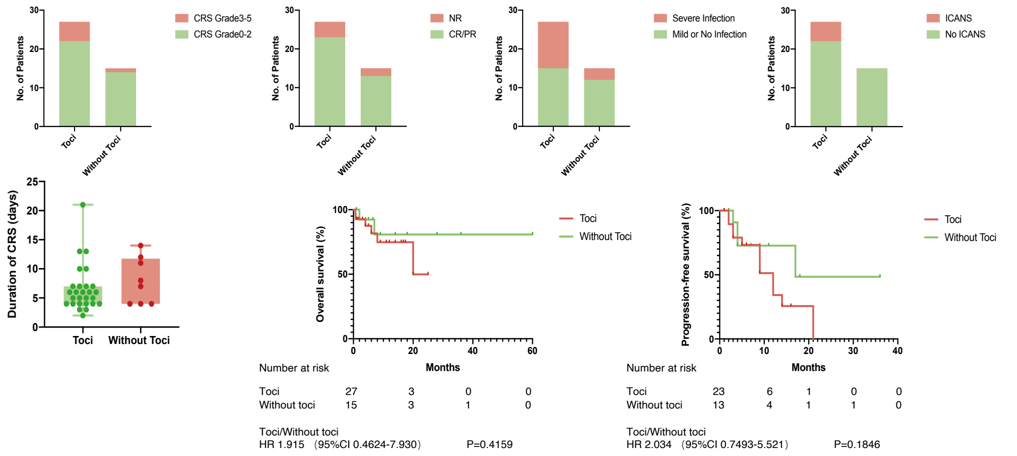
CRS Grade3-5
CRS Grade0-2
NR
CR/PR
Severe Infection
Mild or No Infection
ICANS
No ICANS
No. of Patients
Toci
Without Toci
Duration of CRS (days)
Overall survival (%)
Progression-free survival (%)
Months
Number at risk
Toci 27 3 0 0
Without toci 15 3 1 0
Toci/Without toci
HR 1.915 （95%CI 0.4624-7.930） P=0.4159
Toci 23 6 1 0 0
Without toci 13 4 1 1 0
Toci/Without toci
HR 2.034 （95%CI 0.7493-5.521） P=0.1846

(B)

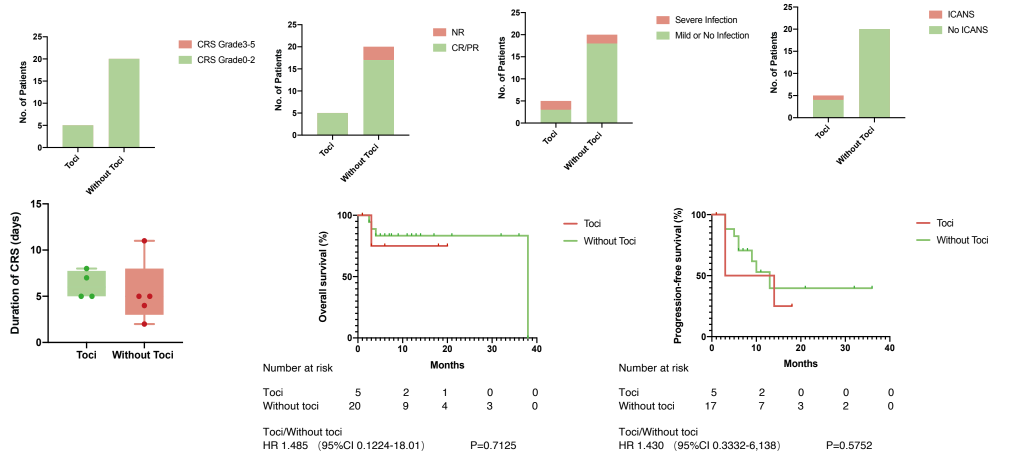
CRS Grade3-5
CRS Grade0-2
NR
CR/PR
Severe Infection
Mild or No Infection
ICANS
No ICANS
No. of Patients
Toci
Without Toci
Duration of CRS (days)
Overall survival (%)
Progression-free survival (%)
Months
Number at risk
Toci 5 2 1 0 0
Without toci 20 9 4 3 0
Toci/Without toci
HR 1.485 （95%CI 0.1224-18.01） P=0.7125
Toci 5 2 0 0 0
Without toci 17 7 3 2 0
Toci/Without toci
HR 1.430 （95%CI 0.3332-6,138） P=0.5752
